# The Repatriation of the Serbians

**Published:** 1918-11-16

**Authors:** Yovan Yovanovitch

**Affiliations:** Serbian Minister, President, Serbian Red Cross Society in Great Britain.


### THE EDITOR'S LETTER-BOX.

**[Correspondence on all subjects is invited, but we cannot in any way be responsible for the opinions expressed by our correspondents, who must give their name and address as a guarantee of good faith, but not necessarily for publication. Correspondents are reminded that brevity of style and conciseness of statement greatly facilitate early insertion.]**

### The Repatriation of the Serbians.

*To the Editor of* The Hospital.

Sir,—May I, through the medium of your paper, appeal to the sympathies of the British public to supplement the generous gifts of the Australian, Canadian, Italian, Japanese, and New Zealand Red Cross Societies to the Serbian Red Cross Society in Great Britain? These gifts are being forwarded to Serbia with all possible dispatch, and will be distributed by the Society's own officers in that country. The loyal and heroic struggle of Serbia for freedom is so well known in Great Britain that I need not recapitulate in detail the tragedy of suffering endured to preserve its national entity and ideals.

The repatriation and rehabilitation of my countrymen must necessarily entail a colossal outlay, and for this reason I appeal to your readers to give to this object whatever assistance is within their power. Contributions should be sent addressed to the Honorary Treasurer, Serbian Red Cross Society in Great Britain, 9 Ennismore Gardens, London, S.W. 7.—Yours faithfully,

Yovan Yovanovitch, Serbian Minister,
President, Serbian Red Cross Society in Great Britain.

### "Is Every Woman a Nurse"?

*To the Editor of* The Hospital.

